# Oral Hygiene Awareness, Practices and Attitudes among Syrian Refugees in Zaatari Camp and Their Impact on Oral Health Status

**DOI:** 10.3290/j.ohpd.b2448601

**Published:** 2021-12-18

**Authors:** Nesreen A. Salim, Faleh A. Sawair, Julian D. Satterthwaite, Zainab Al-Zubi

**Affiliations:** a Associate Professor, Prosthodontics Department, School of Dentistry, The University of Jordan; Consultant, Fixed and Removable Prosthodontics, Jordan University Hospital, Amman, Jordan. Idea, data collection, wrote the manuscript, read and approved the final manuscript.; b Professor, Department of Oral and Maxillofacial Surgery, Oral Medicine and Periodontology, School of Dentistry, The University of Jordan, Jordan University Hospital, Amman, Jordan. Data analysis, co-wrote the manuscript, reviewed, read and approved the final manuscript.; c Professor of Restorative Dentistry, Division of Dentistry, School of Medical Sciences, University of Manchester, Manchester, UK. Co-wrote and critically reviewed the manuscript, read and approved the final manuscript.; d General Dentist, Private Clinic, Amman, Jordan. Contributed to data collection and writing the manuscript, read and approved the final manuscript.

**Keywords:** oral health, oral hygiene habits, refugees, social factors

## Abstract

**Purpose:**

To characterise the oral hygiene habits, attitudes, and oral health practices in relation to sociodemographic factors among refugees in Jordan and to investigate their impact on the oral health status of these refugees.

**Materials and Methods:**

This cross-sectional study consisted of two parts. First, a face-to-face interview was conducted using a structured questionnaire including demographic and oral health-related questions. Second, clinical oral examination was performed using WHO criteria, DMFT and oral health indices (OHI-S). The participants were adults, aged 18 and older. All patients attending dental clinics and accompanying personnel in the waiting areas at Zaatari camp during the study period were invited to participate, with a sample size of 547 refugees (males = 212, females = 335).

**Results:**

547 adult refugees participated. 75.3% reported toothbrushing less than twice daily, while flossing was uncommon (9.5%). Toothbrushing habits were significantly associated with gender and smoking status. Untreated carious lesions had a high incidence (94.1%); the mean number of decayed teeth was 5.4 and was statistically significantly higher in males and smokers. The mean number of missing teeth was 3.2 and was significantly associated with males, age, smoking, and presence of chronic disease. Participants who reported conditions that had persisted 1 year or more and required ongoing medical attention or limited activities of daily living or both (e.g. diabetes mellitus, hypertension, heart diseases, thyroid disease, chronic renal disease, rheumatoid arthritis, anemia, peptic ulcer, or asthma) were recorded as having chronic disease. The mean number of filled teeth was 3.2 and was statistically significantly associated with age and presence of chronic disease. The mean DMFT was 11.8 and was statistically significantly higher in males, older people, smokers, and those with chronic disease. The OHI-S was 2.2. The most common complaint was pain (92.2%), and only 1.1% visited a dentist for a check-up.

**Conclusion:**

The prevalence of caries was extremely high, with poor oral hygiene practices among refugees, justifying the urgent need to develop and implement targeted oral health promotion, preventive programs and curative strategies and to enable collaboration of the oral healthcare providers and funding agencies to design the most appropriate interventions for this disadvantaged population. In addition, this information can be used as a basis upon which preventive programs can be assessed for efficacy.

By mid-March 2011, war and political violence had begun in Syria, following peaceful protests against the government of the Syrian Arab Republic.^28^ By May 2016, there were an estimated 5 million registered Syrian refugees.^27^ The great majority are located in the countries that share land borders with Syria, namely Jordan, Lebanon, and Turkey.^27^ Jordan, a limited-resource developing country, is one of the countries most affected by the Syrian crisis; it accepted a large number of resettlement cases, with the second-highest share of refugees compared to its population.^27^ Al-Zaatari camp, the largest camp in Jordan, is now home to 77,771 refugees.^27^

Refugee oral health is negatively affected by many factors, such as: poor diet and/or nutrition, poverty, the overall burden of resettlement, as well as beliefs, knowledge, and experiences of oral health care.^20-24^ Also, there are restricted resources in the camps, with malnutrition, spread of contagious disease, violence, overcrowding and poor access to clean water being common.^13^ Unmet dental needs are high, with many refugees having never received oral healthcare or basic oral disease prevention.^20,21^ Although the majority of refugees have untreated disease, approximately half of them believe their oral health to be good, very good or excellent.^16^

Extraction due to caries has been shown to be the most common treatment provided for refugees, with endodontic treatment being the least,^19,20^ with limited procedures being provided. There is also a lack of knowledge and awareness, so that refugees do not seek dental treatment unless they suffer pain;^19-22^ the low economic status of this population (who cannot afford private dental care) necessitates public sector dental clinics for dental care, where advanced dental services are not available.

The majority of publications assessing oral health awareness of refugees are from industrialised, developed countries,^12,17,18^ while the majority of refugees are resettled in developing countries (86%).^12^ Thus, little is known about the oral disease status and oral health awareness in Syrian refugees in developing countries.^13,19,20^ Resource-limited countries prioritise prevention and treatment of infectious diseases over oral diseases and non-communicable diseases in general.^13^ To address oral disease at both individual and community levels, oral health awareness needs to be enhanced.^12^ At the same time, further research and baseline information on oral health and habits of refugees in developing countries is required in order to develop and implement appropriate oral health promotion, preventive programs and curative strategies. Thus, the aims of this study were to characterise the oral health habits, perceptions and reasons for seeking oral health care among Syrian refugees in Zaatari camp and to examine their oral health status, none of which have been previously reported. Sociodemographic factors, such as age, gender, education and general health, were considered and included as explanatory factors.

## Materials and Methods

### Ethical Approval

The study was reviewed and approved by the scientific research committee at the Deanship of Academic Research at the University of Jordan, School of Dentistry. Written informed consent was obtained from participants.

### Study Sample

A cross-sectional study was conducted among patients attending the dental clinic at Zaatari refugee camp, east of Mafraq, Jordan. The study was performed in full accordance with all policies of appropriate patient care. Data were collected for a two-month period (between June and August 2019). Participants were adults, aged 18 and older, and all patients attending dental clinics and accompanying personnel in the waiting areas at the camp during the study period were invited to participate. A total of 547 participants (335 females and 212 males) were included in this study. All participants were registered as refugees in Jordan residing in Zaatari camp. Sociodemographic data including age, gender, educational level, smoking, and medical status of the interviewees were recorded. Participants who reported conditions persisting 1 year or more and required ongoing medical attention, and/or who reported limitations of daily activities – e.g. diabetes mellitus, hypertension, heart diseases, thyroid disease, chronic renal disease, rheumatoid arthritis, anemia, peptic ulcer, or asthma – were recorded as having chronic disease. Educational levels were defined as: low (none, primary school), moderate (high school), and high (college and university).

Sample size was determined based on a formula for cross-sectional studies.^26^ As the population of Zaatari camp is known to be 77,771, the following formula was used to determine the required sample size:

sample size = n/1 + [(n-1) / population] = 383

This is the required sample size at a 95% confidence level.

Data collection was planned with a sample size of 547 participants to allow detection of statistically significant differences between subgroups based on gender, age and educational level. The sample distribution was adequate for each category.

### Study Design

The study was divided into two parts. The first part was conducted using a structured questionnaire, and the second part entailed clinical examination of the participants.

#### I: Questionnaire

Data were collected from participants through a face-to-face interview using a structured questionnaire with 12 closed questions. It took an average of 10 to 15 min to complete the questionnaire. The participation rate was 100%; all participants who were invited provided a consent form and agreed to participate in the study. The questionnaire used in this study addressed four aspects:

- Sociodemographic variables: age, gender, educational level, smoking status (current smoker or non-smoker), duration of stay in camp, and medical status.
- Hygiene habits: frequency of toothbrushing, use of dental floss, use of mouthrinses, and the presence of halitosis.
- Dental visits: subjects were asked to report the frequency and reasons for visiting their dentist (before and after coming to the camp).
- Dietary habits, and patient’s satisfaction with the dental/medical services provided in the camp.

Prior to the main study, pilot testing was undertaken with 30 participants in the waiting area of the dental clinics to assess each question for clarity. A printed version of the final questionnaire was used by the study dentist to document participants’ answers. Interviews were conducted by one dentist and another dentist conducted the clinical examination to eliminate any examiner bias in this study.

#### II: Clinical examination

A clinical dental examination was performed by a single examiner experienced in epidemiologic research. A total of 547 participants (335 females, 212 males) were examined. Examinations were conducted in a dental clinic, using a disposable oral mirror and a WHO periodontal probe. Examination was carried out according to the standardised diagnostic criteria outlined by the WHO.^29^ During the consultation, clinical variables were documented on a dental screening form based on the WHO Oral Health Assessment Form.^32^ The clinical examination included the following:

- Decayed, missing, and filled teeth (DMFT) were recorded.
- Oral hygiene status was examined using the simplified oral hygiene index (OHI-S). The examination was performed on the following teeth and surfaces: labial surfaces of teeth 11, 26, 16, 31 and the lingual surfaces of teeth 36 and 46.^8^

The examiner was calibrated for intra-rater reliability by examining a group of 30 patients (not part of the study sample) on two different occasions. Both examinations were then compared, showing an intra-class Cohen’s kappa correlation coefficient of 0.95.

### Statistical Analysis

Statistical analysis was performed using SPSS for Windows v 16.0 (SPSS; Chicago, IL, USA). Descriptive statistics were generated and the chi-squared test, independent samples t-test, and ANOVA were used to examine associations between the different variables. Multivariate logistic and linear regression analyses were then used to control for potential confounding variables and to calculate the odds ratios, coefficients of regression, and 95% confidence intervals for each significant independent variable.The significance level was set at p < 0.05.

## Results

A total of 547 participants (335 females, 61.2%; 212 males, 38.8%) were examined. The sociodemographic characteristics of the participants are shown in Table 1. Females outnumbered males, and most were under 40 years of age with no or only primary school education and with more than 5 years spent in the refugee camp.

**Table 1:** Sociodemographic characteristics of the study population

Variable		Number (%)
Gender	Male	212 (38.8)
	Female	335 (61.2)
Age	18–29	174 (31.8)
	30–39	198 (36.2)
	40–49	106 (19.4)
	≥50	69 (12.6)
Education	None/primary school	335 (61.2)
	High school	137 (25.0)
	College/university	75 (13.7)
Duration of stay in camp (years)	≤5	31 (5.7)
	6	133 (24.3)
	7	263 (48.1)
	≥8	120 (21.9)
Smoking	No	365 (66.7)
	Yes	182 (33.3)
Medical status	Fit	411 (75.1)
	Chronic disease	136 (24.9)

**Fig 1:**
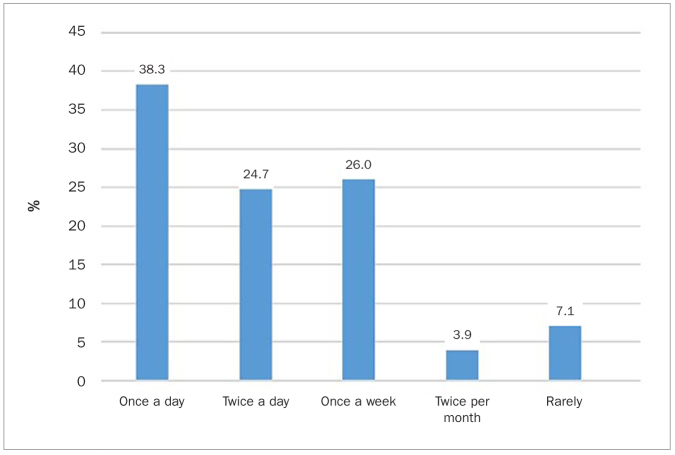
Percentage of adult Syrian refugees who report toothbrushing of different frequencies.

87% reported cleaning their teeth. As shown in Table 2, cleaning of teeth was practiced more frequently by females, young patients, non-smokers, and medically fit patients. In multivariate regression analysis (Table 3), only gender and medical health were found to be independent predictors of cleaning teeth. Females were ca 3.5 times more likely and medically fit patients were ca 2.14 times more likely to clean their teeth compared with males and those with chronic disease, respectively. Of those who cleaned their teeth, 96.4% used a toothbrush, 12.2% rinsed their mouths and gargled, 9.9% used siwak, and 9.5% used dental floss. The use of toothbrush, siwak, and dental floss was not significantly associated with sociodemographic variables. Rinsing and gargling was practiced more often by females and non-smokers, as well as those who had no or only primary school education or university/college education. Multivariate regression analysis (Table 3) showed that only gender and education were found to be independent predictors of rinsing and gargling. Females were about three times more likely to rinse and gargle compared to males. Those with a high-school education were about 75% less likely to rinse and gargle compared with those who had no or primary school education. For those who brushed their teeth, 459 participants, the frequency of toothbrushing is shown in Fig 1. 63% brushed once or twice daily and 37% infrequently. Females and non-smokers brushed their teeth more frequently compared with males (p < 0.01) and smokers (p < 0.01). 62.3% complained of halitosis.

**Table 2:** Percentage of adult Syrian refugees reporting oral hygiene practices according to sociodemographic variables

Variable	Gender		Age				Education			Duration of stay in camp (years)				Smoking		Medical status	
	Male	Female	18–29	30–39	40–49	≥50	None/primary school	High school	College/university	≤5	6	7	≥8	No	Yes	Fit	Chronic disease
Clean their teeth	78.3	92.5	90.8	88.9	83	78.3	88.4	81.8	90.7	90.3	89.5	86.7	84.2	91.5	78	89.3	80.1
p-value	<0.001		<0.05					NS		NS				<0.001		<0.01	
Brush their teeth	94.6	97.4	96.8	96.6	95.5	96.3	95.6	100	94.1	96.4	96.6	96.1	97	94.4	97.3	96.7	95.4
p-value	NS		NS					NS		NS				NS		NS	
Floss their teeth	12.7	7.7	8.2	8.5	12.5	11.1	8.8	7.1	16.2	14.3	5.0	9.6	12.9	10.6	9.0	9.3	10.1
p-value	NS		NS					NS		NS				NS		NS	
Use Siwak	12.7	8.1	10.9	7.6	10.4	13	9.6	8	14.7	16.1	12	8.7	8.3	6.6	11.5	9.7	10.3
p-value	NS		NS					NS		NS				NS		NS	
Rinsing and gargling	6.1	16.1	11.5	12.1	9.4	18.8	15.2	4.4	13.3	16.1	12.8	12.9	9.2	15.1	6.6	11.9	13.2
p-value	<0.01		NS					<0.01		NS				<0.01		NS	
Complaining of halitosis	56.6	66	61.5	68.7	58.5	52.2	63	67.2	50.7	58.1	53.4	66.5	64.2	64.7	57.7	61.1	66.2
p-value	<0.05		NS					NS		NS				NS		NS	
Know that oral health is connected to general health	80.2	75.2	69.5	79.8	85.8	75.4	74	75.9	93.3	74.2	83.5	75.3	75	74	83.5	75.4	82.4
p-value	NS		<0.05					<0.01		NS				<0.05		NS	
Think that regular visits to the dentist are essential	72.2	76.7	73	75.3	79.2	72.5	75.2	71.5	80	80.6	72.2	76.4	73.3	77.5	69.8	73.7	78.7
p-value	NS		NS					NS		NS				NS		NS	
Satisfied with the medical services provided at the camp’s clinics	17.9	21.8	23	19.2	18.9	18.8	22.7	17.5	14.7	32.3	18	17.1	26.7	21.9	17	22.1	14.7
p-value	NS		NS					NS		NS				NS		NS	

NS: not significant.

**Table 3:** Stepwise logistic regression modelling of sociodemographic variables on reported oral hygiene practices/attitudes and knowledge about link between oral and general health.

Attitudes and oral hygiene practices	Variable	Regression coefficient	p-value	Odds ratio	Confidence level for odds ratio
Clean their teeth	Gender*	1.252	<0.001	3.50	2.07–5.92
	Medical health^$^	0.759	0.006	2.14	1.25–3.67
Rinsing and gargling	Gender*	1.091	0.001	2.98	1.57–5.65
	Education^ǂ^		0.009		
	High school	-1.352	0.002	0.26	0.11–0.62
	College/university	0.007	0.985	1.01	0.48–2.12
Know that oral health is connected to general health	Smoking^#^	0.541	0.021	1.72	1.08–2.73
	Education^ǂ^		0.005		
	High school	0.116	0.62	1.12	0.71–1.79
	College/university	1.56	0.001	4.76	1.86–12.20

*Male as reference category. $ Having chronic disease as reference category. ^ǂ^Primary school as reference category. # Non-smoker as reference category.

The majority (77.1%) knew that oral health is connected to general health, particularly those who were 40–49 years of age, had university/college education, and were smokers. Multivariate regression analysis (Table 3) showed that only smoking and education were found to be independent predictors of this knowledge. Smokers were 1.7 times and those with a college/university education were 4.76 times more likely to be aware of this connection, compared with nonsmokers and those with no or primary school education, respectively.

**Fig 2:**
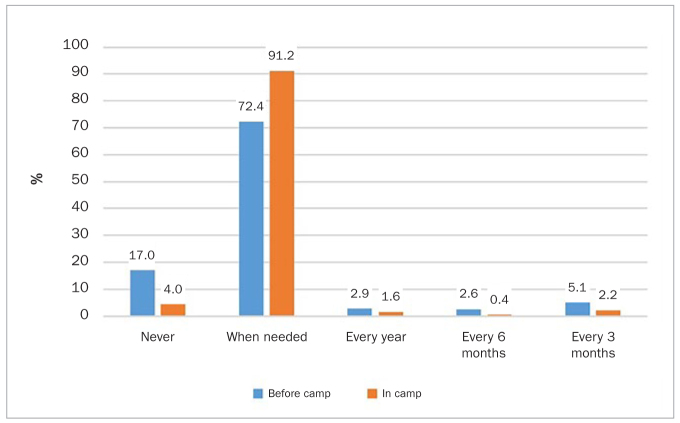
Percentage of adult Syrian refugees who report visiting dentists before and after coming to the camp.

The frequency of visiting the dentist before and after coming to the camp is shown in Fig 2. Before coming to the camp, 17% had never visited a dentist, compared to only 4.6% after they came to camp. Before coming to the camp, those who were 18-29 years of age and those who were medically fit (p = 0.008 and p < 0.01, respectively) visited a dentist less frequently; while in camp, the frequency of visiting a dentist was not associated with sociodemographic variables.

**Fig 3:**
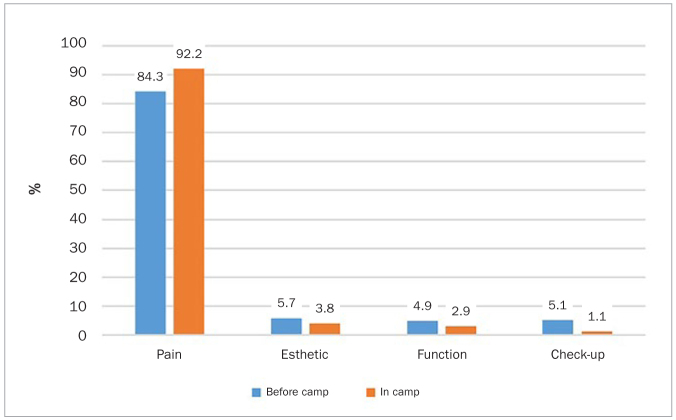
The reasons reported by adult Syrian refugees for visiting dentists before and after coming to the camp.

The reasons for dental visits before and after coming to the camp are presented in Fig 3. The percentage of participants who only visited a dentist due to pain was high after coming to camp (p < 0.001). Three quarters of patients thought that regular dental visits were essential (Table 2). Only 20.3% of the study sample were satisfied with the medical services provided at the camp’s clinics.

When asked about dietary composition, 58.5% reported carbohydrates, 5.3% reported fatty foods, 7.1% reported sugary foods, 6.2% reported meats, and 22.9% reported vegetables as the highest component of their diet. Females reported higher intake of carbohydrates and less fatty foods compared with males (0.015, p < 0.05), and smokers reported a higher intake of meats and fewer vegetables compared with non-smokers (p < 0.01).

The DMFT score, plaque index, and calculus index and its association with sociodemographic variables are shown in Tables 4 and 5. 94.1% had untreated carious lesions. The mean number of decayed teeth was 5.4 ± 3.7, and was statistically significantly higher in males and smokers. In multivariate linear regression (Table 5), smoking was the only independent predictor of the number of decayed teeth. Smokers had an increase of 1.29 in their mean number of decayed teeth compared to nonsmokers.

**Table 4:** Series of bivariate analyses of sociodemographic variables on oral health indices

Variable	Gender		Age				Education			Duration of stay in camp (years)				Smoking		Medical status	
	Male	Female	18–29	30–39	40–49	≥50	None/primary school	High school	College/university	≤5	6	7	≥8	No	Yes	Healthy	Chronic disease
Number of decayed teeth (mean ±SD)	5.84 ± 4.21	5.16 ± 3.28	5.64 ± 3.53	5.48 ± 3.48	5.30 ± 4.30	4.93 ± 3.62	5.56 ± 3.64	5.48 ± 3.81	4.72 ± 3.57	5.42 ± 3.36	5.24 ± 3.50	5.42 ± 3.81	5.65 ± 3.70	5.00 ± 3.23	6.29 ± 4.33	5.48 ± 3.59	5.27 ± 3.96
p-value	<0.05		NS					NS		NS				<0.001		NS	
Number of missing teeth (mean ± SD)	3.8 ± 4.00	2.81 ± 3.12	2.28 ± 2.50	2.78 ± 2.53	4.47 ± 4.18	4.72 ± 5.50	3.18 ± 3.69	3.153.07	3.35 ± 3.51	2.32 ± 2.37	3.25 ± 3.19	3.22 ± 3.66	3.31 ± 3.77	2.86 ± 3.90	3.86 ± 3.26	2.83 ± 3.22	4.30 ± 4.12
p-value	<0.01		<0.001					NS		NS				<0.01		<0.001	
Number of filled teeth (mean ± SD)	2.86 ± 3,60	3.423.47	2.37 ± 3.03	3.13 ± 3.07	4.34 ± 4.50	3.78 ± 3.71	3.05 ± 3.17	3.24 ± 4.13	3.83 ± 3.81	3.90 ± 3.68	3.60 ± 3.89	3 ± 3.31	3.03 ± 3.51	3.22 ± 3.46	3.18 ± 3.66	2.86 ± 3.17	4.24 ± 4.28
p-value	NS		<0.001					NS		NS				NS		<0.001	
DMFT score	12.51 ± 6.31	11.39 ± 5.58	10.28 ± 5.10	11.39 ± 5.23	14.11 ± 6.50	13.44 ± 7.13	11.79 ± 6.00	11.87 ± 6.10	11.89 ± 5.02	11.64 ± 5.26	12.09 ± 5.54	11.64 ± 5.97	11.99 ± 6.32	11.08 ± 5.43	13.32 ± 6.49	11.17 ± 5.35	13.81 ± 6.95
p-value	<0.05		<0.001					NS		NS				<0.001		<0.001	
Plaque index	1.59 ± 0.71	1.27 ± 0.55	1.21 ± 0.55	1.45 ± 0.65	1.49 ± 0.62	1.55 ± 0.74	1.42 ± 0.64	1.39 ± 0.64	1.28 ± 0.61	1.51 ± 0.68	1.36 ± 0.60	1.41 ± 0.65	1.36 ± 0.65	1.30 ± 0.58	1.58 ± 0.71	1.36 ± 0.61	1.49 ± 0.71
p-value	<0.001		<0.001					NS		NS				<0.001		NS	
Calculus index	1.41 ± 0.75	1.07 ± 1.11	1.08 ± 1.41	1.20 ± 0.71	1.25 ± 0.69	1.46 ± 0.75	1.22 ± 0.72	1.23 ± 1.55	1.10 ± 0.76	1.34 ± 0.88	1.24 ± 1.54	1.20 ± 0.75	1.13 ± 0.69	1.11 ± 1.09	1.39 ± 0.73	1.19 ± 1.06	1.24 ± 0.78
p-value	<0.001		NS					NS		NS				<0.01		NS	

p-values of independent sample t-test and ANOVA. NS: not significant.

**Table 5:** Linear regression analysis modelling of sociodemographic variables on reported oral hygiene practices/attitudes

Oral health indices	Variable	Non-standardized Coefficients (B)	Standardized Coefficients (β)	95% confidence interval for B	p-value
Number of decayed teeth	Smoking	1.294	0.166	0.65-1.94	<0.001
Number of missing teeth	Age	0.758	0.216	0.45-1.06	<0.001
	Gender	0.858	0.119	0.28-1.44	0.004
	Medical health	0.815	0.100	0.11-1.52	0.024
Number of filled teeth	Age	0.477	0.135	0.16-0.79	0.003
	Medical health	0.961	0.118	0.24-1.68	0.009
DMFT score	Age	1.007	0.171	0.50-1.52	<0.001
	Smoking	1.981	0.158	0.97-2.99	<0.001
	Medical health	1.618	0.119	0.44-2.80	0.007
Plaque index	Gender	0.236	0.181	0.11-0.36	<0.001
	Age	1.00	0.157	0.05-0.15	<0.001
	Smoking	0.138	0.102	0.01-0.27	0.035
Calculus index	Gender	0.343	0.168	0.17-0.51	<0.001

The mean number of missing teeth was 3.2 ± 3.6, and was statistically significantly higher in males, older age groups, those with a history of smoking, and those with chronic systemic disease. Multivariate linear regression (Table 5) showed only age, gender and medical health to be statistically significant predictors of the number of missing teeth. There was an average increase of 0.76 (range 0.45 to 1.06) in the number of missing teeth when each age group was compared with the younger age group. Males had an average of 0.86 (range 0.28 to 1.44) and those with chronic systemic disease had an average of 0.82 (range 0.11 to 1.52) increase in number of missing teeth compared with females and medically fit patients, respectively.

The mean number of filled teeth was 3.2 ± 3.6 and was statistically significantly associated with increasing age and presence of chronic systemic disease. There was an average increase of 0.48 (range 0.16 to 0.76) in the number of filled teeth when each age group was compared with the younger age group, and those with chronic systemic disease had an average increase of 0.96 (range 0.24 to 1.68) in number of filled teeth compared with medically fit patients.

The mean DMFT score was 11.8 ± 5.9 and was statistically significantly higher in males, older age groups, smokers, and those with chronic systemic disease. Multivariate linear regression showed only age, smoking and medical health to be statistically significant predictors of DMFT score. There was an average increase of 1.01 (range 0.50 to 1.52) in DMFT when each age group was compared with the younger age group. Smokers had an average of 1.98 (range 0.97 to 2.99) and those with chronic systemic disease had an average increase of 1.62 (range 0.44 to 2.80) in DMFT compared with nonsmokers and medically fit patients, respectively.

The mean simplified debris index (DI-S) was 1.4 ± 0.6, and was statistically significantly associated with male gender, older age groups, and a history of smoking. There was an average increase of 1.00 (range 0.05 to 0.15) in DI-S when each age group was compared with the younger age group. Males had an average increase of 0.24 (range 0.11 to 0.36) and smokers had an average increase of 0.14 (range 0.01 to 0.27) in DI-S compared with females and nonsmokers, respectively. The mean simplified calculus index (CI-S) was 1.2 ± 1.0 and was statistically significantly higher in males, and smokers, however, multivariate analysis showed that only gender was a statistically significant independent predictor of CI-S; males had an average increase of 0.34 (range 0.17 to 0.51) in CI-S compared with females. A statistically significant association was found between frequency of brushing and DMFT mean score (p < 0.001), frequency of brushing and mean plaque index (p < 0.001), and between frequency of brushing and mean calculus index (p < 0.001). No statistically significant association was found between diet and mean DMFT score.

## Discussion

Refugees suffer high rates of dental disease, which has a substantial effect on their quality of life and poses a high economic burden on the health-care system in the host countries.^1,6^ To our knowledge, no studies have been published on the oral health of refugees, their oral hygiene habits, dental decay, oral hygiene indices, and the reason for and pattern of visiting a dentist before and after settling in the Zaatari refugee camp. The present study highlights the high prevalence of caries, with untreated carious lesions and poor oral hygiene among refugees. This justifies the urgent development and implementation of targeted oral health promotion, preventive programs and curative strategies, and will enable collaboration between oral healthcare providers and funding agencies to design the most appropriate interventions for this disadvantaged population. In addition, this information can be used as a basis on which preventive programs can be assessed for efficacy.^3^

Most participants clean their teeth, but with less than one tenth using dental floss. However, oral hygiene practices were generally weak, with most participants using a toothbrush to clean their teeth less often than twice a day. These results compared unfavourably with other refugees studies.^3,12^ A previous study showed that self-reported poor oral hygiene (never/rarely brushed) was associated with an increased risk of cardiovascular disease: these data underscore the importance of maintaining a good oral hygiene among patients.^5^ The majority of refugees believed in the relationship between oral and general health, although this was mainly reported by educated participants, in contrast to the poor oral health practices and beliefs of less educated participants, who made up the majority.

The universal occurrence of dental calculus suggests inadequate oral hygiene practices and unhealthy dental behaviours.^7^ In this study, the OHI-S (DI+CI) was noticeably higher than previously reported results for refugees in Canada.^9^ Sociodemographic factors including age, gender, smoking and education were statistically significantly associated with the attitudes and status of oral health among refugees. Males, smokers and medically compromised and older participants presented more missing teeth and poorer oral hygiene. The few previous studies examining adult refugees have shown trends similar to those of our study. In this context, a previous study^25^ used the Community Periodontal Index (CPI) to evaluate periodontal health and oral hygiene, and found a statistically significant increase in calculus presence with age, while females showed statistically significantly lower levels of calculus compared to males, who had higher CPI scores.

In this study, a high DMFT mean score was noted, markedly higher than a comparable age group in Germany.^25^ High mean DMFT scores have been reported for non-refugee populations (11.2);^10^ however, this is due to a high number of filled teeth, whereas in our study, the high DMFT of refugess relates to a high number of untreated caries. Females, educated persons, and non-smokers showed a statistically significantly higher mean number of filled teeth, fewer missing teeth and better oral hygiene compared to males and less educated participants in this study. Similar to our results, other studies have reported that females use dental services more regularly than males.^7^ Similarly, a study published in 1996^2^ found an increase in the mean of both DMFT and OHI-S scores in then-Yugoslavian and Moroccan refugees with age,^2^ and the DMFS (decayed, missing and filled surfaces) index was reported to be statistically significantly higher in older and less educated refugees.^15^

The high prevalence of untreated caries in refugees may be attributed to limited access to oral health care, mainly due to shortage or unavailability of dental professionals and oral hygiene utensils.^19,20^ Additionally, the process of migration and adapting to a new culture can influence the utilisation of dental services, with low priority often being placed on dental care during migration.^12^ It is important for decision makers in the host country to target this at-risk population as early on as possible. This could be achieved through providing access to immediate oral assessment and treatment upon arrival. Likewise, it is important to establish active inclusion of refugees in the existing health structures and implementing community-based care and prevention programs for this population, as well as curative treatments.^20,24^

Unexpectedly, in this study, the percentage of participants who never visited a dentist in their home country was higher than those who never visited the dentist in the camp. This could be due to the availability of free dental services in the camp compared to the home country.^21^ However, the overall dental-visit frequency was less in the camp. Additionally, a few people reported visiting the dentist every 1–2 years (only 1.6% of individuals in the camp and 2.9% in their home country), despite 75% of them thinking that regular dental visits are essential. This contradictory attitude could be related to long waiting lists, unavailability of care when needed, or the long time to complete treatment (it has been estimated to take twice as long to complete the same treatment procedure in this population/setting).^13^ Additionally, waiting-room environments may have an impact, and oral health behaviour is also influenced by poor engagement with administrative staff at care facilities, past experiences, and lack of dental insurance.^3^ Further insight in relation to engagement/attendance was provided by a study of Pakistani refugees in Australia – their preference was for accessing a dentist in their home country, suggesting greater trust in dental practitioners in their home country, a better understanding of the health system there, and perhaps greater affordability of services.^18^ Other contributing factors were limited access, unfamiliarity with the new health-care system, social isolation, language barriers, as well as generally low emphasis on oral health promotion during the resettlement period under such conditions.

In our study, the most common reason for visiting the dentist was pain (92%). A regular check-up as a reason for a dental visit was rarely reported. Possible reasons include lack of awareness about the availability of dental treatment clinics in the camp and the limited number of dental clinics in Zaatari camp (only seven dental clinics for approximately 80,000 residents).^14^ Moreover, at present, the basic dental services provided in the Zaatari camp are limited to tooth extractions and, to a limited extent, basic restorative procedures, which are provided mainly by NGOS and volunteer services.^3,13,25^ This could explain why 80% of the participants in this study were not satisfied with the services provided in the camp.

The study setting presented some practical challenges. The lack of radiographic diagnostic aids (e.g. bitewings) presented a potential limitation in relation to diagnosis of approximal caries, and this may have resulted in underestimating caries prevalence. Although the Community Periodontal Index (CPI) may be used to assess periodontal status/treatment need in detail, the aim of this study was to evaluate the overall oral hygiene status, and OHI-S was used to achieve this. OHI-S has been shown to correlate more closely with inflammatory indices in adults,^11^ which suggests its particular applicability for examinations in this age group.^4,11^ Moreover, the CPI increases the costs of evaluation.^17^ This cross-sectional analysis provided insight into the oral health of this population at only one point in time. Nonetheless, this research is the first to document the oral health status of adult Syrian refugees and their oral health needs, and address some of the obstacles they face.

## Conclusions

This study provides an understanding of the problems and concerns regarding the poor oral health and poor oral hygiene practices of refugees. Dental care needs of this underprivileged refugee community are largely unmet. In order to improve the oral health status of such groups, rather than relying solely on limited resources to satisfy immediate urgent needs, increased focus must be placed on the use of community-based refugee oral health promotion, with tailored preventive health services, and appropriate advice on how to access oral health care in a timely manner.

A number of public health interventions have been suggested to tackle the burden of oral diseases amongst Syrian refugee children.^23^ They include a combination of measures that aim to reduce sugar intake, improve diet and oral hygiene, increase access to an appropriate source of fluoride, and fortify the capacity of the Syrian dental workforce to deal with the growing burden of caries in this underprivileged population. However, the provision of healthcare for refugee populations can be a challenge for host nations, particularly those countries with limited resources that are overwhelmed by an influx of refugees. There is also a need to train health professionals who are often faced with limited resources and are unfamiliar with working in such environments, the challenges of a dense refugee population, remote location of the refugee camps, as well as concomitant health issues and a tendency to prioritise other, more urgent, health needs. Good understanding and collaboration between authorities and community organisations is required. For example, collaboration with dental/medical schools at major universities/hospitals in a mutually beneficial fashion would allow students to meet academic requirements by treating refugees, whilst providing a solution to the issues of treatment budgets and availability of services and facilities to provide these treatments, especially beyond basic primary healthcare.
